# Supplementary material for: Food Insecurity Is Associated with Diet Quality in Pregnancy: A Cross-Sectional Study
Source: Nutrients. 2024 Apr 28;16(9):1319. doi: 10.3390/nu16091319 (PMC11085356; doi:10.3390/nu16091319)
Supplement: Supplementary file 1 [file nutrients-16-01319-s001.zip › FigureS1.pdf]

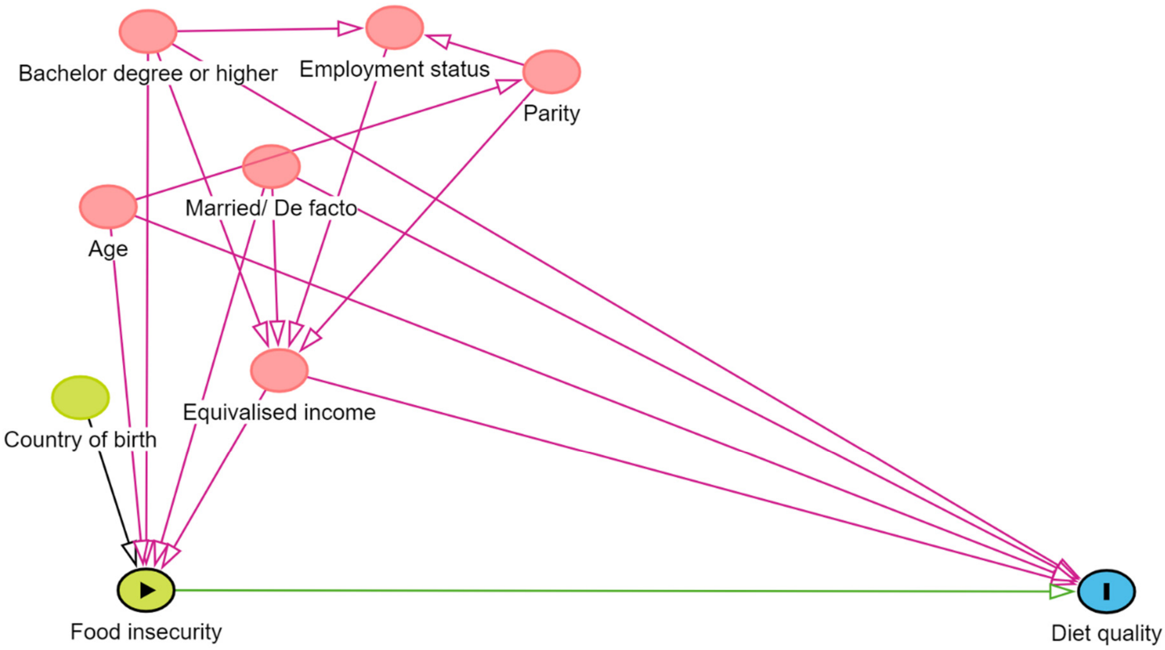

Figure S1: Directed acyclic graph[a] visualising the assumed relationship between household food insecurity and prenatal diet quality. [a]Simplified version for clarity. Exposure variable: household food insecurity; Outcome variable: prenatal diet quality; Pink nodes: potential confounding factors; Green nodes: ancestor of the exposure.
