# Supplementary material for: Food Insecurity Is Associated with Diet Quality in Pregnancy: A Cross-Sectional Study
Source: Nutrients. 2024 Apr 28;16(9):1319. doi: 10.3390/nu16091319 (PMC11085356; doi:10.3390/nu16091319)
Supplement: Supplementary file 1 [file nutrients-16-01319-s001.zip › FigureS2.pdf]

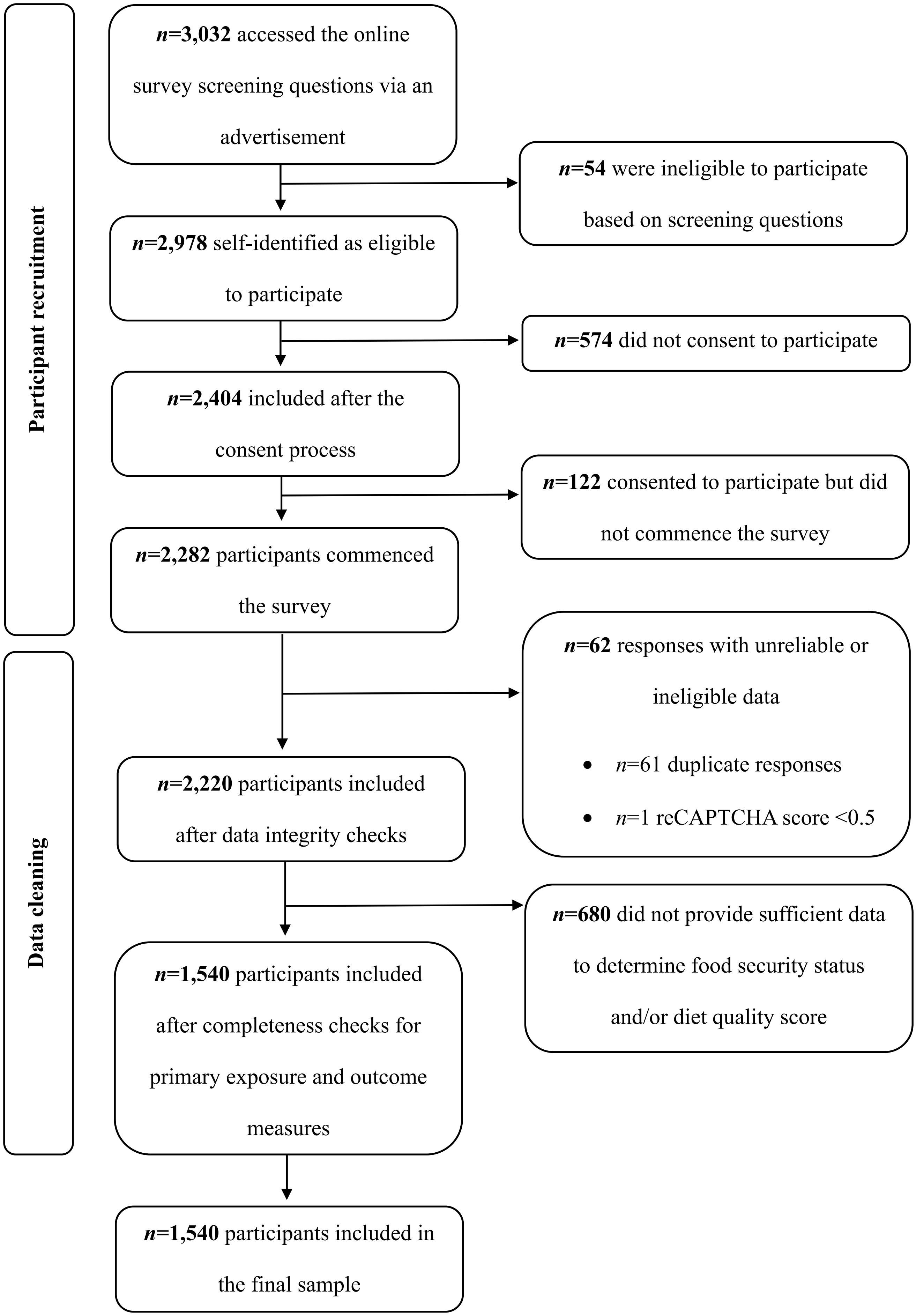
Participant recruitment
Data cleaning
n=3,032 accessed the online survey screening questions via an advertisement
n=54 were ineligible to participate based on screening questions
n=2,978 self-identified as eligible to participate
n=574 did not consent to participate
n=2,404 included after the consent process
n=122 consented to participate but did not commence the survey
n=2,282 participants commenced the survey
n=62 responses with unreliable or ineligible data
• n=61 duplicate responses
• n=1 reCAPTCHA score <0.5
n=2,220 participants included after data integrity checks
n=680 did not provide sufficient data to determine food security status and/or diet quality score
n=1,540 participants included after completeness checks for primary exposure and outcome measures
n=1,540 participants included in the final sample

Figure S2: Flow diagram summarising online survey participation between August 2022 and March 2023 by pregnant women in Australia and eligibility for inclusion in the analytic sample (*n*=1,540).
