# Supplementary material for: Food Insecurity Is Associated with Diet Quality in Pregnancy: A Cross-Sectional Study
Source: Nutrients. 2024 Apr 28;16(9):1319. doi: 10.3390/nu16091319 (PMC11085356; doi:10.3390/nu16091319)
Supplement: Supplementary file 1 [file nutrients-16-01319-s001.zip › TableS2_CHERRIES.pdf]

Table S2: The Checklist for Reporting Results of Internet E-Surveys (CHERRIES) [1]

Item Category	Checklist Item(s)	Checklist Item(s) Explanation	Page Number	Additional Information
Design	Describe survey design	Describe target population, sample frame. Is the sample a convenience sample? (In "open" surveys this is most likely.)	3	
IRB (Institutional Review Board) approval and informed consent process	IRB approval<br>Informed consent<br>Data protection	Mention whether the study has been approved by an IRB.<br>Describe the informed consent process. Were the participants told the length of time of the survey, which data were stored and where and for how long, who the investigator was, and the purpose of the study?	3	A participant information statement was provided to respondents before the consent process. This listed the study investigators and discussed the purpose of the survey and the use, storage and protection of personal information and data.

Item Category	Checklist Item(s)	Checklist Item(s) Explanation	Page Number	Additional Information
		If any personal information was collected or stored, describe what mechanisms were used to protect unauthorised access.		Participants were then asked if they consented to take part and were required to click 'Yes, I consent' to confirm their consent before proceeding to the online survey.
Development and pre-testing	Development and testing	State how the survey was developed, including whether the usability and technical functionality of the electronic questionnaire had been tested before fielding the questionnaire.	3-5	The survey was developed using pre-existing scales to assess food security status, usual dietary intake, and diet quality score.<br><br>The survey was pilot tested among five colleagues and/or people similar to the target group (i.e. women of

Item Category	Checklist Item(s)	Checklist Item(s) Explanation	Page Number	Additional Information
				reproductive age or pregnant women) and feedback was requested on the length of time it took to complete, whether there were any questions that were difficult to understand and/or answer, or any questions that raised concerns.<br><br>The functionality of the survey (e.g. adaptive questioning) was also tested by members of the research team and colleagues.
Recruitment process and	Open survey versus closed survey	An "open survey" is a survey open for each visitor of a site, while a closed survey is	3	The survey was open (password not required to enter the survey).

Item Category	Checklist Item(s)	Checklist Item(s) Explanation	Page Number	Additional Information
description of the sample having access to the questionnaire	Contact mode<br>Advertising the survey	only open to a sample which the investigator knows (password-protected survey).<br>Indicate whether or not the initial contact with the potential participants was made on the Internet. (Investigators may also send out questionnaires by mail and allow for Web-based data entry.)<br>How/where was the survey announced or advertised? Some examples are offline media (newspapers), or online (mailing lists – If yes, which ones?) or banner ads (Where were these banner ads posted and what did they look like?). It is important to know the		Advertisements were displayed physically at antenatal clinics and electronically via Facebook and Instagram. Advertisements stated that ‘we’re running an important study to understand how difficulty making ends meet relates to diet quality in pregnancy’ and/or asked participants to ‘help us learn how we can support fair access to food for all women/families during pregnancy by participating in this research study’. Advertisements also stated ‘to thank

Item Category	Checklist Item(s)	Checklist Item(s) Explanation	Page Number	Additional Information
		wording of the announcement as it will heavily influence who chooses to participate. Ideally, the survey announcement should be published as an appendix.		you for participating in the survey, we are offering entry into a draw to win 1 of 3 $200 gift cards' or similar.
Survey administration	Web/e-mail<br>Context<br>Mandatory/voluntary<br>Incentives<br>Time/date<br>Randomization of items or questionnaires	State the type of e-survey (e.g., one posted on a Web site, or one sent out through e-mail). If it is an e-mail survey, were the responses entered manually into a database, or was there an automatic method for capturing responses?<br>Describe the website (for mailing list/newsgroup) in which the survey was posted. What is the website about, who is	3-4	The survey was voluntary, with participants having to click on the advertisement or scan a QR code to obtain entry. Entry in a prize drawing (randomly selected) to win one of three AUD$200 gift cards was offered.

Item Category	Checklist Item(s)	Checklist Item(s) Explanation	Page Number	Additional Information
	Adaptive questioning<br>Number of items<br>Number of screens (pages)<br>Completeness check<br>Review step	visiting it, what are visitors normally looking for? Discuss to what degree the content of the website could pre-select the sample or influence the results. For example, a survey about vaccination on an anti-immunisation website will have different results from a web survey conducted on a government website<br>Was it a mandatory survey to be filled in by every visitor who wanted to enter the website, or was it a voluntary survey?<br>Were any incentives offered (e.g., monetary, prizes, or non-monetary		Items were not randomised. Adaptive questioning was used.<br><br>The number of items per page varied. A maximum of 244 questions over 35 web pages were asked (this was reduced by adaptive questioning for some respondents).<br><br>Most questions were mandatory to respond to (with an option to click 'don't know' or 'prefer not to say'), with the respondents unable to move forward in the survey without

Item Category	Checklist Item(s)	Checklist Item(s) Explanation	Page Number	Additional Information
		incentives such as an offer to provide the survey results)?<br>In what timeframe were the data collected?<br>To prevent biases, items can be randomised or alternated.<br>Use adaptive questioning (certain items, or only conditionally displayed based on responses to other items) to reduce the number and complexity of the questions.<br>What was the number of questionnaire items per page? The number of items is an important factor for the completion rate.<br>Over how many pages was the questionnaire distributed? The number of		completing them. However, there were some questions that were not mandatory. For these questions, participants received a reminder message to prompt them to answer them before continuing to the next question.<br><br>Respondents were able to click the "Back" button, enabling them to change their responses. Incomplete surveys could be continued by the respondent for up to one week from last response/action. After one week

Item Category	Checklist Item(s)	Checklist Item(s) Explanation	Page Number	Additional Information
		items is an important factor for the completion rate.<br>It is technically possible to do consistency or completeness checks before the questionnaire is submitted. Was this done, and if "yes", how (usually JAVAScript)? An alternative is to check for completeness after the questionnaire has been submitted (and highlight mandatory items). If this has been done, it should be reported. All items should provide a non-response option, such as "not applicable" or "rather not say", and selection of one response option should be enforced.		with no further response/action from the respondent, the data in the incomplete survey was automatically recorded by Qualtrics and the participant could no longer edit or continue that response.

Item Category	Checklist Item(s)	Checklist Item(s) Explanation	Page Number	Additional Information
		State whether respondents were able to review and change their answers (e.g., through a Back button or a Review step which displays a summary of the responses and asks the respondents if they are correct).		
Response rates	Unique site visitor view rate (ratio of unique survey visitors/unique site visitors)<br>Participation rate (Ratio of unique visitors who agreed to participate/unique	If you provide view rates or participation rates, you need to define how you determined a unique visitor. There are different techniques available, based on IP addresses, cookies or both.<br>Requires counting unique visitors to the first page of the survey, divided by the number of unique site visitors (not page views!). It	6; Supplementary Materials Figure S2	Paid Meta advertising resulted in 3,210 clicks and a total of 177,856 impressions.<br><br>Of the survey respondents who consented and commenced the survey (*n*=2,220), 92.5% were recruited via paid Meta advertising, 5% were recruited via unpaid Facebook

Item Category	Checklist Item(s)	Checklist Item(s) Explanation	Page Number	Additional Information
	first survey page visitors)	is not unusual to have view rates of less than 0.1 % if the survey is voluntary.		advertisements, and 2.2% were recruited via flyers at local public hospitals.
	Completion rate (Ratio of users who finished the survey/users who agreed to participate)	Count the unique number of people who filled in the first survey page (or agreed to participate, for example by checking a checkbox), divided by visitors who visit the first page of the survey (or the informed consents page, if present). This can also be called "recruitment" rate. The number of people submitting the last questionnaire page, divided by the number of people who agreed to participate (or submitted the first survey page). This is only relevant if there is a separate		In the analytic sample (*n*=1,540), 92% were recruited via paid Meta advertising, 5.2% were recruited via unpaid Facebook advertisements, and 2.5% were recruited via flyers at local public hospitals.

Item Category	Checklist Item(s)	Checklist Item(s) Explanation	Page Number	Additional Information
		"informed consent" page or if the survey goes over several pages. This is a measure for attrition. Note that "completion" can involve leaving questionnaire items blank. This is not a measure for how completely questionnaires were filled in. (If you need a measure for this, use the word "completeness rate".)		
Preventing multiple entries from the same individual	Cookies used<br>IP check<br>Log file analysis<br>Registration	Indicate whether cookies were used to assign a unique user identifier to each client computer. If so, mention the page on which the cookie was set and read, and how long the cookie was valid. Were duplicate entries avoided by preventing users access to the	3-4	IP address was used to flag potential duplicate responses. Those responses were then reviewed manually to determine if they were duplicates (including review of contact information, sociodemographic

Item Category	Checklist Item(s)	Checklist Item(s) Explanation	Page Number	Additional Information
		survey twice; or were duplicate database entries having the same user ID eliminated before analysis? In the latter case, which entries were kept for analysis (e.g., the first entry or the most recent)?<br>Indicate whether the IP address of the client computer was used to identify potential duplicate entries from the same user. If so, mention the period of time for which no two entries from the same IP address were allowed (e.g., 24 hours). Were duplicate entries avoided by preventing users with the same IP address access to the survey twice; or were duplicate database entries having		variables, and identification of any other suspicious information), as it is possible for the same IP address to be recorded for different people. If duplicates were identified, the most complete response was kept (i.e. if there was one response where the participant dropped out early in the survey and one response where they completed the full survey, the complete response was retained). If both responses were complete, the most recent response was retained.

Item Category	Checklist Item(s)	Checklist Item(s) Explanation	Page Number	Additional Information
		the same IP address within a given period of time eliminated before analysis? If the latter, which entries were kept for analysis (e.g., the first entry or the most recent)? Indicate whether other techniques to analyze the log file for identification of multiple entries were used. If so, please describe. In “closed” (non-open) surveys, users need to login first and it is easier to prevent duplicate entries from the same user. Describe how this was done. For example, was the survey never displayed a second time once the user had filled it in, or was the		Other checks for duplicates included review of contact information data. The survey was not a closed survey.

Item Category	Checklist Item(s)	Checklist Item(s) Explanation	Page Number	Additional Information
		username stored together with the survey results and later eliminated?<br>If the latter, which entries were kept for analysis (eg, the first entry or the most recent)?		
Analysis	Handling of incomplete questionnaires<br>Questionnaires submitted with an atypical timestamp<br>Statistical correction	Were only completed questionnaires analyzed? Were questionnaires which terminated early (where, for example, users did not go through all questionnaire pages) also analyzed?<br>Some investigators may measure the time people needed to fill in a questionnaire and exclude questionnaires that were submitted too soon. Specify the timeframe that was	5-6; Supplementary Materials Figure S2	Incomplete questionnaires were analysed, if the participant had provided sufficient data for the main exposure (food security status) and outcome (diet quality score) variables in this study.<br><br>Survey completion times were checked to identify people who

Item Category	Checklist Item(s)	Checklist Item(s) Explanation	Page Number	Additional Information
		used as a cut-off point, and describe how this point was determined.<br>Indicate whether any methods such as weighting of items or propensity scores have been used to adjust for the non-representative sample; if so, please describe the methods.		completed the survey >2 standard deviations below the median duration and less than one-third of the median duration. None were identified.<br><br>Methods such as weighting of items or propensity scores were not used.

References

1. Eysenbach, G. Improving the quality of web surveys: The Checklist for Reporting Results of Internet E-Surveys (CHERRIES). *J. Med. Internet Res.* **2004**, *6*, e34, doi:10.2196/jmir.6.3.e34.
