# Supplementary material for: Food Insecurity Is Associated with Diet Quality in Pregnancy: A Cross-Sectional Study
Source: Nutrients. 2024 Apr 28;16(9):1319. doi: 10.3390/nu16091319 (PMC11085356; doi:10.3390/nu16091319)
Supplement: Supplementary file 1 [file nutrients-16-01319-s001.zip › TableS3.pdf]

Table S3: Dietary Guidelines Index 2013 (DGI-13) criteria [1], modified for pregnancy

DGI-13 Component	Description/Indicator	Maximum Score	Criteria for Maximum Score	Criteria for Minimum Score
**1. Enjoy a wide variety of nutritious foods**	Dietary variety: proportion of food from each of the five core food groups eaten at least one serve per week	10	100%	0%
**2. Vegetables**	Servings of vegetables per day	10	≥5	0
**3. Fruit**	Servings of fruit per day	10	≥2	0
**4. Grain (cereal) foods**	Servings of grains/cereals per day	5	≥8.5[a]	0
	Mostly wholegrain or high fibre cereals: Type of bread usually consumed	5	Wholemeal/ high fibre bread	White bread
**5. Lean meat and poultry, fish, eggs, nuts and seeds and legumes/beans**	Servings of lean meats and alternatives per day	5	≥3.5[a]	0
	Proportion of lean meats and alternatives to total meat and alternatives per day	5	100%	0%

**6. Milk, yoghurt, cheese and/or their alternatives**	Servings of dairy and alternatives per day	10	≥2.5	0
**7. Drink plenty of water**	Total beverage intake per day (cups)	5	≥9[b]	0
	Proportion of water to total beverage intake per day	5	≥50%	0%
**8. Limit intake of foods containing saturated fat, added salt, added sugars and alcohol**	Servings of discretionary foods per day	10	≤2.5	>2.5
**9. Limit intake of foods high in saturated fat**	Trimming of fat from meat	5	Usually	Never or rarely
	Type of milk usually consumed	5	Skim, low fat, or reduced fat milk	Whole / full cream milk
**10. Small allowance of unsaturated oils, fats or spreads**	Servings of unsaturated oils, fats, and spreads per day	10	≤2	>2
	Salt added during cooking	5	Never or rarely	Usually

**11. Limit intake of foods and drinks containing added salt**	Salt added after cooking/during the meal	5	Never or rarely	Usually
**12. Limit intake of foods and drinks containing added sugars**	Servings of foods and drinks containing added sugars per day	10	≤1.25	>1.25
**13. Not drinking alcohol is the safest option in pregnancy**[a]	Alcohol intake in this pregnancy	10	None[a]	Any

[a]Modified to reflect recommendations for pregnancy as per the Australian Dietary Guidelines [2].

[b]Modified to reflect fluid recommendations in pregnancy [3].

References

1. Thorpe, M.G.; Milte, C.M.; Crawford, D.; McNaughton, S.A. A revised Australian dietary guideline index and its association with key sociodemographic factors, health behaviors and body mass index in peri-retirement aged adults. *Nutrients* **2016**, *8*, 160, doi:10.3390/nu8030160.
2. National Health and Medical Research Council. Australian Dietary Guidelines. **2013**.
3. National Health and Medical Research Council. Nutrient Reference Values for Australia and New Zealand. Available online: https://www.nrv.gov.au/ (accessed on August 21, 2023).
