# Supplementary material for: Food Insecurity Is Associated with Diet Quality in Pregnancy: A Cross-Sectional Study
Source: Nutrients. 2024 Apr 28;16(9):1319. doi: 10.3390/nu16091319 (PMC11085356; doi:10.3390/nu16091319)
Supplement: Supplementary file 1 [file nutrients-16-01319-s001.zip › TableS4.pdf]

Table S4: Sociodemographic characteristics of the analytic sample of 1,540 pregnant women in Australia who participated in an online survey, compared to survey respondents who dropped out and/or did not provide sufficient data to be included in the study

	Mean (SD[a]) or *n* (%)		
	**Analytic sample** (*n*=1,540)	**Respondents unable to be included in analytic sample[b]**	***P* value[c]**
**Age** (years)	30.6 (4.5)	29.7 (4.9)[d]	<0.001
**Trimester**			0.307
First	307 (19.9)	154 (22.6)	
Second	703 (45.6)	293 (43.1)	
Third	530 (34.4)	233 (34.3)	
**Pregnancy type**			0.212
Singleton	1516 (98.4)	672 (98.8)	
Twins	24 (1.6)	7 (1.0)	
Triplets or more	0 (0)	1 (0.1)	
**Pregnancy care**			0.518
Public	1073 (71.2)	475 (72.5)	
Private	435 (28.8)	180 (27.5)	
Missing	32	25	
**Previous births**			0.619
0	855 (55.5)	365 (53.7)	
1	462 (30.0)	218 (32.1)	

>1	223 (14.5)	97 (14.3)	
**Children in household**			0.088
No	817 (53.1)	269 (48.8)	
Yes	723 (46.9)	282 (51.2)	
Missing	0	129	
**Born in Australia**			0.964
No	267 (17.5)	114 (17.6)	
Yes	1260 (82.5)	535 (82.4)	
Missing	13	31	
**Aboriginal and/or Torres Strait Islander**			0.073
No	1495 (97.1)	625 (95.6)	
Yes	45 (2.9)	29 (4.4)	
Missing	0	26	
**Married / De facto**			0.101
No	105 (6.9)	58 (8.9)	
Yes	1427 (93.1)	596 (91.1)	
Missing	8	26	
**Bachelor degree or higher**			<0.001
No	621 (40.4)	323 (49.2)	
Yes	918 (59.6)	333 (50.8)	
Missing	1	24	
**Household income (AUD[e])**			<0.001
$0-$25,999	54 (3.6)	27 (5.9)	

$26,000-$51,999	110 (7.3)	56 (12.3)	
$52,000-$103,999	415 (27.7)	142 (31.1)	
$104,000-$207,999	705 (47.1)	180 (39.4)	
≥$208,000	214 (14.3)	52 (11.4)	
Missing	42	223	
**Household income (AUD[e])**			<0.001
<$104,000 per year	579 (38.7)	225 (49.2)	
≥$104,000 per year	919 (61.3)	232 (50.8)	
Missing	42	223	
**Low SES[f] area**			0.002
No (IRSAD[g] deciles 4-10)	1216 (79.2)	407 (72.7)	
Yes (IRSAD[g] deciles 1-3)	319 (20.8)	153 (27.3)	
Missing	5	120	
**Smoking status**			0.464
Non-smoker	1503 (97.6)	626 (97.1)	
Smoker (any frequency)	37 (2.4)	19 (2.9)	
Missing	0	35	
**Pre-pregnancy BMI[h] category**			0.662
<18.5	52 (3.5)	20 (3.6)	
18.5-24.9	720 (48.6)	267 (47.8)	
25.0-29.9	372 (25.1)	131 (23.4)	
≥30.0	337 (22.8)	141 (25.2)	
Missing	59	121	

[a]SD: Standard deviation.

[b]Maximum of *n*=680. *N* varies for each variable, depending on when respondents dropped out of the survey.

[c]*P* values derived from an independent samples *t*-test for age (continuous variable) and χ^2 tests or Fisher's exact tests (if assumptions for χ^2 test were violated) for all other variables (categorical).

[d]*n*=662

[e]AUD: Australian dollars.

[f]SES: Socioeconomic status.

[g]IRSAD: Index of Relative Socioeconomic Advantage and Disadvantage. Deciles were derived from self-reported residential postcode, as an indicator of area-level socioeconomic status. Lower deciles reflect higher relative socioeconomic disadvantage and lower relative advantage in the area (vice versa for higher deciles).

[h]BMI: Body mass index.
