# Supplementary material for: Food Insecurity Is Associated with Diet Quality in Pregnancy: A Cross-Sectional Study
Source: Nutrients. 2024 Apr 28;16(9):1319. doi: 10.3390/nu16091319 (PMC11085356; doi:10.3390/nu16091319)
Supplement: Supplementary file 1 [file nutrients-16-01319-s001.zip › TableS5.pdf]

Table S5: Sensitivity analysis (removal of outliers) for adjusted[a] associations of household food insecurity severity and diet quality, as measured by total Dietary Guideline Index 2013 (DGI-13) score, in a sample of 1,540 pregnant women who participated in an Australian online survey

	**Original adjusted[a] model** (*n*=1,492)		**Sensitivity analysis[b]** (*n*=1,490)[b]	
	Adjusted R^2: 9.9%		Adjusted R^2: 10.3%	
	**Adjusted[a] β (95% CI[c])**	***P* value**	**Adjusted[a] β (95% CI[c])**	***P* value**
**High food security**	Reference		Reference	
**Marginal food security**	-1.9 (-3.7, -0.1)	0.036	-1.8 (-3.6, -0.1)	0.044
**Low food security**	-3.6 (-5.9, -1.3)	0.002	-3.5 (-5.7, -1.2)	0.003
**Very low food security**	-5.3 (-7.7, -2.8)	<0.001	-5.3 (-7.8, -2.9)	<0.001

[a]Adjusted for age, education, equivalised household income, and relationship status. *N* for models lower than total sample size (*n*=1,540) due to missing data for covariates.

[b]*n*=2 outliers (standardised residuals >3 SD) removed.

[c]CI: Confidence interval.
