# Supplementary material for: Food Insecurity Is Associated with Diet Quality in Pregnancy: A Cross-Sectional Study
Source: Nutrients. 2024 Apr 28;16(9):1319. doi: 10.3390/nu16091319 (PMC11085356; doi:10.3390/nu16091319)
Supplement: Supplementary file 1 [file nutrients-16-01319-s001.zip › TableS6.pdf]

Table S6: Regression modelling for associations of household food insecurity[a] with odds of meeting fruit intake recommendations in a sample of 1,540 pregnant women who participated in an Australian online survey

**Model 1** (*n*=1,540)		**Model 2** (*n*=1,540)[b]		**Model 3** (*n*=1,539)[c]		**Model 4** (*n*=1,498)[d]		**Model 5** (*n*=1,532)[e]		**Model 6** (*n*=1,492)[f]	
Nagelkerke R^2: 0.036		Nagelkerke R^2: 0.042		Nagelkerke R^2: 0.056		Nagelkerke R^2: 0.038		Nagelkerke R^2: 0.039		Nagelkerke R^2: 0.061	
**OR[g] (95% CI[h])**	***P* value**	**AOR[b,i] (95% CI[h])**	***P* value**	**AOR[c,i] (95% CI[h])**	***P* value**	**AOR[d,i] (95% CI[h])**	***P* value**	**AOR[e,i] (95% CI[h])**	***P* value**	**AOR[f,i] (95% CI[h])**	***P* value**
0.51 (0.41-0.62)	<0.001	0.54 (0.44-0.67)	<0.001	0.61 (0.49-0.76)	<0.001	0.55 (0.44–0.70)	<0.001	0.53 (0.43-0.65)	<0.001	0.64 (0.50-0.81)	<0.001

[a]Reference group is high food security (food secure). Food security status was dichotomised (marginal, low, and very low food security collapsed to form the food insecure group).

[b]Adjusted for age.

[c]Adjusted for education. *N* lower due to missing data for this variable.

[d]Adjusted for equivalised household income. *N* lower due to missing data for this variable.

[e]Adjusted for relationship status. *N* lower due to missing data for this variable.

[f]Adjusted for age, education, equivalised household income, and relationship status. *N* lower due to missing data for these variables.

[g]OR: Odds ratio (unadjusted).

[h]CI: Confidence interval.

[i]AOR: Adjusted odds ratio.
