# Supplementary material for: Food Insecurity Is Associated with Diet Quality in Pregnancy: A Cross-Sectional Study
Source: Nutrients. 2024 Apr 28;16(9):1319. doi: 10.3390/nu16091319 (PMC11085356; doi:10.3390/nu16091319)
Supplement: Supplementary file 1 [file nutrients-16-01319-s001.zip › TableS8.pdf]

Table S8: Regression modelling for associations of household food insecurity[a] with odds of meeting both fruit and vegetable intake recommendations in a sample of 1,540 pregnant women who participated in an Australian online survey

**Model 1** (*n*=1,540)		**Model 2** (*n*=1,540)[b]		**Model 3** (*n*=1,539)[c]		**Model 4** (*n*=1,498)[d]		**Model 5** (*n*=1,532)[e]		**Model 6** (*n*=1,498)[f]	
Nagelkerke R^2: 0.031		Nagelkerke R^2: 0.032		Nagelkerke R^2: 0.064		Nagelkerke R^2: 0.034		Nagelkerke R^2: 0.031		Nagelkerke R^2: 0.068	
**OR[g] (95% CI[h])**	***P* value**	**AOR[b,i] (95% CI[h])**	***P* value**	**AOR[c,i] (95% CI[h])**	***P* value**	**AOR[d,i] (95% CI[h])**	***P* value**	**AOR[e,i] (95% CI[h])**	***P* value**	**AOR[f,i] (95% CI[h])**	***P* value**
0.31 (0.14-0.67)	0.003	0.31 (0.14-0.69)	0.004	0.45 (0.20-1.00)	0.051	0.41 (0.18-0.94)	0.036	0.31 (0.14-0.68)	0.004	0.54 (0.23-1.23)	0.141

[a]Reference group is high food security (food secure). Food security status was dichotomised (marginal, low, and very low food security collapsed to form the food insecure group) due to low cases meeting both the fruit and vegetable recommendations, which limited power.

[b]Adjusted for age.

[c]Adjusted for education. *N* lower due to missing data for this variable.

[d]Adjusted for equivalised household income. *N* lower due to missing data for this variable.

[e]Adjusted for relationship status. *N* lower due to missing data for this variable.

[f]Adjusted for education and equivalised household income. *N* lower due to missing data for these variables.

[g]OR: Odds ratio (unadjusted).

[h]CI: Confidence interval.

[i]AOR: Adjusted odds ratio.
